# Nanostructured Materials for Water Purification: Adsorption of Heavy Metal Ions and Organic Dyes

**DOI:** 10.3390/polym14112183

**Published:** 2022-05-27

**Authors:** Won San Choi, Ha-Jin Lee

**Affiliations:** 1 Department of Chemical and Biological Engineering, Hanbat National University, 125 Dongseodaero, Yuseong-gu, Daejeon 34158, Korea; choiws@hanbat.ac.kr; 2 Division of Chemistry and Bio-Environmental Sciences, Seoul Women’s University, 621 Hwarangro, Nowon-gu, Seoul 01797, Korea

**Keywords:** heavy metal adsorption, organic dye adsorption, nanostructured materials, wastewater treatment

## Abstract

Chemical water pollution poses a threat to human beings and ecological systems. The purification of water to remove toxic organic and inorganic pollutants is essential for a safe society and a clean environment. Adsorption-based water treatment is considered one of the most effective and economic technologies designed to remove toxic substances. In this article, we review the recent progress in the field of nanostructured materials used for water purification, particularly those used for the adsorption of heavy metal ions and organic dyes. This review includes a range of nanostructured materials such as metal-based nanoparticles, polymer-based nanomaterials, carbon nanomaterials, bio-mass materials, and other types of nanostructured materials. Finally, the current challenges in the fields of adsorption of toxic materials using nanostructured materials are briefly discussed.

## 1. Introduction

With continuous industrialization, water pollution has led to severe environmental crises around the world and has been a challenging issue to solve. Water contamination not only damages the environment, but also poses a serious threat to human health and ecosystems. Moreover, the demand for fresh water is continually increasing with the growth of the human population and the rise in living standards. However, water contaminants reduce the freshwater supply provided by both surface-water and groundwater resources [1,2,3]. In addition, due to their limited availability, naturally available freshwater reserves are still unable to meet this demand. The World Meteorological Organization (WMO) has reported that, by 2050, more than 5 billion people worldwide will have inadequate access to water [4]. This problem can only be overcome if people find other ways to reserve or generate fresh water, or convert wastewater into a usable form. To address these issues, various water purification methods and novel materials have been developed.

**Figure 1:**
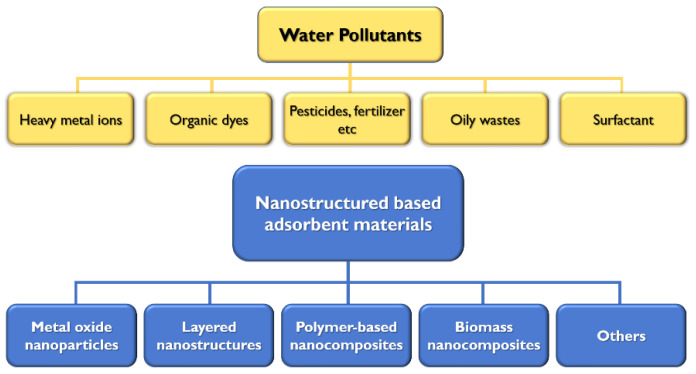
Various water pollutants and nanostructured materials used for the removal of pollutants.

Nanomaterial is defined as material with an external dimension, an internal structure, or a surface structure at the nanoscale [5]. Nanoscale materials often exhibit very different physical or chemical properties compared with larger size materials. In particular, the large surface area and high surface free energy of nanomaterials often result in a high density of active sites per unit mass, resulting in improved surface reactivity [6,7,8,9]. Because of the abundance of active sites along with their thermal and mechanical stabilities, nanomaterials have been actively used as water treatment materials to purify water by removing pollutants such as heavy metals, toxic organic dyes, oily waste, and various industrial and agricultural waste [10,11,12,13,14,15,16]. Various sources of water pollution and nanostructured materials used for their purification are shown in Figure 1.

These water treatment processes might be significantly enhanced by incorporating nanomaterials within the water purification system, taking advantage of their remarkable capabilities. However, there are drawbacks to using nanomaterials directly in water treatment. First, nanomaterials, particularly nanoparticles, tend to agglomerate during the water purification process due to their high surface areas and strong dipole–dipole interactions, resulting in significant loss of activity [17,18,19]. In addition, isolating nanomaterials from aqueous media remains a difficult task, as it may result in the unintended leakage of nanomaterials into ecosystems, posing a significant risk to the environment and human health [20,21,22]. Therefore, cost-effective and environmentally friendly nanostructure-based materials designed to be fixed onto various matrices to prevent nanocomposite aggregation have been developed for water treatment purposes [23,24,25,26,27,28,29,30,31,32,33]. Although a variety of nanomaterials for water purification have been reported and show great potential, most have several disadvantages and thus do not meet the conditions necessary for practical use.

Herein, we review the recent progress of nanostructured materials and their application in water purification, especially the adsorption of toxic materials such as heavy metals and organic dyes. In each section, we describe some representative results and explain the concepts, the nanostructured materials employed, their preparation methods, and their effects on the water purification process. Finally, challenges in the field of toxic material adsorption using nanostructured materials are also discussed.

## 2. The Adsorption of Heavy Metals and Organic Dyes

Industrial waste released into water containing toxic organic dyes and heavy metal ions from the textile, leather, printing, paper, and electroplate industries is a potential hazard to ecological environments and poses serious risks to human health and other living organisms [34,35,36,37,38]. Heavy metal ions are non-biodegradable and tend to accumulate in living organisms. Therefore, removing heavy metal ions from wastewater, including lead, mercury, cadmium, arsenic, and chromium, is crucial for protecting human health and the environment [39,40]. Meanwhile, most organic dyes are toxic and have a significant influence on photosynthetic activity in aquatic biota. In particular, dyes composed of aromatic rings are inert and non-biodegradable. When they are discharged into wastewater, they are carcinogenic and mutagenic to humans through contaminated drinking water [41,42]. Due to their limited biodegradability, dyes are generally extracted from an aqueous medium, and a number of technologies designed to eliminate them from wastewater have been developed.

Various methods for removing heavy metal ions and dyes from wastewater treatment include ion exchange, chemical precipitation, bio-sorption, filtration, reverse osmosis, and adsorption [43,44,45,46,47,48,49]. Among these techniques, adsorption is recognized as an effective and economic method [50,51]. Adsorption is not only one of the most efficient ways to dramatically reduce the release of toxic substances, but it also immobilizes functional molecules for catalysis or other applications [36,52]. The most crucial prerequisite for a good adsorbent is a large interface [52]. Therefore, porous and nanostructured materials with significantly improved surface area are widely used for potential adsorption [53,54,55].

### 2.1. Removal of Water Contaminants by Adsorption

There are two types of adsorption: physical adsorption and chemisorption [56]. Physical adsorption is a process in which van der Waals force attraction or dispersion causes an adsorbate to attach to an adsorbent surface. Physical adsorption is characterized by a relatively low enthalpy of adsorption and the adsorbed layer may vary in thickness from monolayer to multilayer [57]. It occurs more readily than chemisorption at room temperature, and adsorbed layers are more readily desorbed. On the other hand, chemisorption involves the formation of ions or covalent bonds by chemical reactions, and forms a monolayer only. This is characterized by difficulty in desorbing the adsorbed layer [55,57]. Commonly, physical adsorption occurs in a second layer on top of the chemisorbed material in the first layer [58].

#### 2.1.1. Adsorption Capacity

Batch adsorption experiments under various operating conditions, such as pH, ionic strength, temperature, etc., are generally performed to evaluate the adsorption capacity of adsorbents [30,32]. The adsorption capacity is the most important parameter for evaluating an adsorbent. Here, the adsorption capacity (*q_e_*, mg g^−1^) and efficiency (*E*, %) were calculated using the following equations [36]:

(1) $$q_{e}=\frac{\left(C_{0}-C_{e}\right)\times V}{m}$$

(2) $$E\left(\%\right)=\frac{\left(C_{0}-C_{e}\right)}{C_{0}}\times 100$$

where *C*_0_ (mg L^−1^) and *C_e_* (mg L^−1^) are the initial and equilibrium concentrations of the adsorbates, respectively; *V* (*V*) is the solution volume, and *m* (g) is the mass of the adsorbent. *C_e_* (mg L^−1^) of heavy metal ions can be determined by an atomic absorption spectroscopy (AAS) [30] or inductively coupled plasma mass spectroscopy (ICP-MS) [28]. The concentration of residual organic dyes can be determined by UV-vis spectroscopy at the maximum absorption wavelength, using a measured extinction coefficient from Beer’s law analysis of each dye solution [51].

#### 2.1.2. Adsorption Isotherms

Theoretical adsorption capacity can be calculated using adsorption isotherm models [3]. The adsorption behavior of contaminants by nanomaterials are described by two isothermal models, the Langmuir and Freundlich models. These models are expressed using the following equations [59,60]:

(3) $$\mathrm{Langmuir}:\;\frac{C_{e}}{q_{e}}=\frac{1}{q_{m}K_{L}}+\frac{C_{e}}{q_{m}}$$

(4) $$\mathrm{Freundlich}:\;\ln q_{e}=\frac{1}{n}\ln C_{e}+\ln K_{F}$$

where *Ce* (mg L^−1^) is the equilibrium concentration of the heavy metal ions; *q_e_* (mg g^−1^) is the equilibrium adsorption capacity of the heavy metal ions adsorbed on the adsorbent; *q_m_* (mg g^−1^) is the maximum adsorption capacity of the adsorbents; *K_L_* (L mg^−1^) and *K_F_* (mg g^−1^) are the Langmuir and Freundlich constants, respectively, representing the adsorptive capacity and the affinity between the adsorbate and the adsorbent; *n* is the constant related to the heterogeneity of the adsorbent sites and indicates the affinity between the adsorbate and the adsorbent. The Langmuir isotherm model is commonly used for monolayer adsorption in which most of the adsorption sites have identical affinities toward the adsorbate, whereas the Freundlich isotherm model is used to describe a heterogeneous chemisorption process in which the surface is not energetically uniform [61].

#### 2.1.3. Adsorption Kinetics

Observation of adsorption kinetics is essential in adsorption studies, because it provides a prediction of adsorption rate and the possible adsorption mechanism. The adsorption kinetics of aqueous heavy metals by nanomaterials are generally well-fitted by the pseudo-first-order (PFO), pseudo-second-order (PSO), Elovich, and intra-particle diffusion models, which are described as follows [62]:

pseudo-first-order: ln(*q_e_* − *q_t_*) = ln(*q_e_*) − *k*_1_·*t* (5)

(6) $$\mathrm{pseudo}\text{-}\mathrm{second}\text{-}\mathrm{order}:\;\frac{t}{q_{t}}=\frac{1}{k_{2}\cdot q_{e}^{2}}+\frac{1}{q_{e}}t$$

(7) $$\mathrm{Elovich}:\;q_{t}=\frac{1}{\beta }\ln\left(\alpha \beta \right)+\frac{1}{\beta }\ln t$$

(8) $$\mathrm{Intra}\text{-}\mathrm{particle}\;\mathrm{diffusion}:\;q_{t}=k_{ip}t^{1/2}+C$$

where *q_e_* (mg g^−1^) and *q_t_* (mg g^−1^) are the adsorption capacity at equilibrium and at contact time *t* (min), respectively; *k*_1_ (min^−1^), *k*_2_ (g mg^−1^ min^−1^) and *k_ip_* (g mg^−1^ min^−1/2^) are the equilibrium rate constant of the PFO, PSO, and intra-particle diffusion models, respectively; *C* (mg g^−1^) is the intercept that approximates the thickness of the boundary layer [63]. The PSO and PFO models are based on the assumption that the adsorption rate is controlled by chemical adsorption, which includes electron transfer and sharing between the adsorbate and the adsorbent [64]. The Elovich model is a chemical-reaction-based adsorption reaction model [62,64]. On the other hand, the intra-particle diffusion kinetic model shows that the sorption process is diffusion-controlled if its rate is dependent on the rate at which the adsorbate and the adsorbent diffuse towards one another [65].

#### 2.1.4. Thermodynamic Studies

Thermodynamic studies of the adsorption are indispensable for understanding adsorption reactions such as spontaneity and internal energy transfer. Changes in thermodynamic parameters, Δ*G*^0^ (Gibbs free energy, kJ mol^−1^), Δ*H*^0^ (average enthalpy, kJ mol^−1^), and Δ*S*^0^ (standard entropy, kJ mol^−1^ K^−1^) can be determined from temperature-dependent adsorption isotherms. The parameters are calculated using the following equations [66,67]:

(9) $$\Delta G^{0}=-RT\ln K_{d}$$

(10) $$lnK_{d}=\frac{\Delta S^{0}}{R}-\frac{\Delta H^{0}}{RT}$$

where *R* (8.314 J mol^−1^ K^−1^) is the gas constant; *T* (K) is the absolute temperature; *K_d_* (L g^−1^) is the thermodynamic equilibrium constant, which is calculated by plotting ln(*q_e_/C_e_*) versus *q_e_* and extrapolating *q_e_* to zero.

### 2.2. Adsorbent Materials

Recently, various nanomaterial-based adsorbents for the removal of heavy metal ions and dyes have been investigated, including nanosized metal oxides, carbon-based nanomaterials, biomass, polymers, and other materials. These emerging materials, which have shown desirable characteristics for heavy metal and dye adsorption, are discussed in the following sections.

#### 2.2.1. Inorganic Nanostructured Materials

- Metal Oxide Nanoparticles

Due to their large surface areas and high activities, metal oxide nanoparticles (MONs), such as iron oxides, magnesium oxides, titanium oxides, manganese oxides, and aluminum oxides, are classified as promising for targeting contaminants in water systems [10,68,69,70]. Magnetic MONs, in particular, are gaining intensive attention because they can easily be isolated from water when exposed to a magnetic field [71,72,73,74,75,76]. Magnetic MON-based adsorbents also facilitate recycling or regeneration from aqueous solutions. This ease of separation is critical for enhancing operational efficiency and lowering costs during water treatment. However, the surface energy of metal oxides increases as their size is reduced from the micrometer to the nano scale, resulting in a decrease in their stability [77,78]. As a result, MONs are agglomerated due to a strong van der Waals force [79,80], and their water treatment efficiency can dramatically decrease. In recent years, much work has been done to functionalize magnetic MONs by coating them with polymers, carbon, inorganic materials, etc., to overcome aggregation and the resulting limited contaminant adsorption capacity [80,81,82,83].

**Figure 2:**
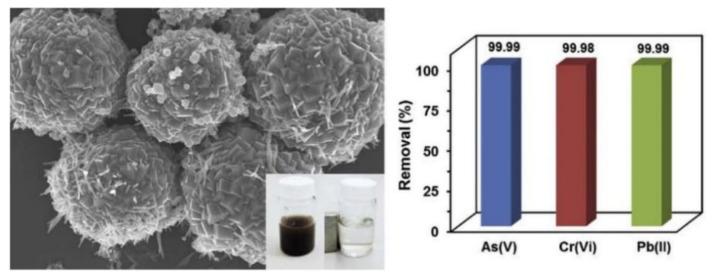
(**Left**) SEM image of hierarchical structured IO@CaCO_3_ adsorbent consisting of magnetic iron oxide nanoneedles. (**Right**) As(V), Cr(VI) and Pb(II) heavy metal ion removal efficiencies of IO@CaCO_3_ adsorbents. Reproduced with permission from [84]. Copyright 2017 Elsevier.

Islam et al. reported a hierarchically structured IO@CaCO_3_ adsorbent consisting of magnetic iron oxide (IO) nanoneedles and mesoporous calcite (CaCO_3_), for the rapid purification of water contaminated by heavy metal ions such as Cr(VI), As(V) and Pb(II) to drinking water level (Figure 2) [84]. The hierarchical structure of the IO@CaCO_3_ adsorbent was characterized by FE-SEM and spherical particles with a diameter of 2–4 μm, with nanosized IO nanoneedles inside the particles. They reported that the IO@CaCO_3_ adsorbent, due to the synergistic effect of needle-like IO and porous CaCO_3_, removed both anionic (As(V) and Cr(VI)) and cationic (Pb(II)) heavy metal ions from aqueous solutions, with significantly enhanced adsorption capacities (184.1, 251.6, and 1041.9 mg g^−1^, for As(V), Cr(VI), and Pd(II), respectively) compared to conventional adsorbents. Furthermore, it was demonstrated that it can easily be separated by an external magnetic field, allowing economical reuse, and that it has an excellent heavy metal removal capability. Wei et al. reported hollow nest-like *α*-Fe_2_O_3_ nanostructures for water treatment, prepared by a simple and green synthesis route using a microwave-assisted, template-free, hydrothermal method [85]. They showed that the hollow nest-like α-Fe_2_O_3_ nanospheres with mesoporous structures consist of hierarchical spheres assembled of nanorod subunits, and exhibit excellent adsorption capacities toward As(V) (75.3 mg g^−1^) and Cr(VI) (58.5 mg g^−1^) ions and Congo red (160 mg g^−1^) that are much more enhanced than those of most reported nanomaterial-based adsorbents. Wang et al. investigated silica-coated Fe_3_O_4_ magnetic nanospheres (Fe_3_O_4_@SiO_2_) for the high removal of Congo red (CR) dye from wastewater [86]. The calculated adsorption capacity of Fe_3_O_4_@SiO_2_ for CR was 54.64 mg g^−1^, and the adsorption mechanism was dominated by an electrostatic interaction between CR and the adsorbent. 

**Figure 3:**
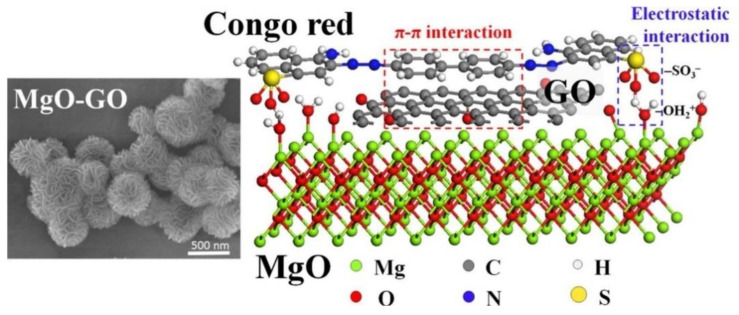
(**Left**) FE-SEM images of samples MgO-GO. (**Right**) Schematic illustration of the adsorption mechanism between MgO-GO composite and Congo red, which involves electrostatic and π–π interactions. Reproduced with permission from [90]. Copyright 2018 Elsevier.

MgO has also eceived considerable attention in toxic wastewater treatment, as it is considered a nontoxic, cost-effective, and eco-friendly material [87,88,89]. Bai et al. showed that porous rod-like MgO nanoparticles have an extremely high adsorption capacity with respect to Congo red dye (3236 mg g^−1^), through a simple precipitation reaction between Mg^2+^ and carbonate (CO_3_^2−^) in the presence of a trace amount of Na_2_SiO_3_ [88]. Cao et al. investigated flower-like MgO nanoparticles with a high surface area, using a microwave-assisted solvothermal process for heavy metal removal [89]. The flower-like MgO nanoparticles showed an adsorption capacity of 1980 and 1980 mg/g for Pb(II) and Cd(II), respectively. They reported that the adsorption mechanism involves a solid–liquid interfacial cation exchange between magnesium and lead or cadmium cations, resulting in a significantly high absorption capacity. Xu et al. synthesized hierarchical MgO microspheres with a diameter of 500 nm using a solvothermal process [90]. According to FE-SEM observation, the MgO microspheres maintained their microsphere shape after forming a composite with graphene oxide and showed a large specific surface area and pore volume. Due to the large specific area, the hierarchical microspheres exhibited a large adsorption capacity (237.0 mg g^−1^) for Congo red (Figure 3).

- Layer Double Hydroxide(LDH)-Based Materials

Layered double hydroxides (LDHs) are known as hydrotalcite materials and have been classified as two-dimensional anionic clays, which can be represented by [M^2+^_1−*x*_M^3+^*_x_*(OH)_2_]*^x^*^+^[(A^n−^)*_x/n_*]*^x^*^−^·*m*H_2_O (M^2+^ and M^3+^ are divalent and trivalent metals, respectively; A^−^ is the interlayer anion) [91,92]. The uniquely layered structure, together with the chemical composition of the inorganic layers and the interlayer anions, offers tremendous potential for dispersing and tuning active sites at the atomic level [93,94,95]. Furthermore, a simple synthetic protocol allows micro- to nanoscale manipulation of the LDH structure to stabilize active sites in the LDH layer or the interlayer during a chemical process. LDH and its related materials have received substantial attention for their use in the adsorption of anionic and cationic pollutants, due to their highly tunable interior architecture, exchangeable interlayer ions, large surface area, low cost, and absence of toxicity. In particular, spherical LDH micro- or nanoparticles with porous architectures have attracted attention due to their structural stability and high surface area, both of which are essential factors enhancing their removal capabilities for water contaminants [36,96,97,98,99].

Mubarak et al. prepared Mg/Fe-LDH hollow nanospheres with a high specific surface area by a one-step thermal method in an ethylene glycol solution containing two metal precursors, Mg^2+^ and Fe^3+^ [36]. They further synthesized its oxidized form into Mg/Fe-layered double oxide (Mg/Fe-LDO) nanospheres by thermal calcination, and used them to purify wastewater contaminated with oxyanionic heavy metals ions such as AsO_4_^3−^ and Cr_2_O_7_^2−^ or anionic organic dyes such as Congo red and methyl blue. The Mg/Fe-LDO nanospheres showed maximum adsorption capacities of 178.6 mg g^−1^ for AsO_4_^3−^ and 148.7 mg g^−1^ for Cr_2_O_7_^2−^. Complete removal (~99.9%) from wastewater, to a level appropriate for potable water (based on WHO standards), was achieved within 10–20 min. In addition, these Mg/Fe-LDO nanospheres exhibited over 99% removal efficiency in less than 5 min for methyl blue and 25 min for Congo red. The maximum adsorption capacities for methyl blue and Congo red were 2000 and 1250 mg g^−1^ at room temperature, respectively. Such a high adsorption efficiency for the heavy metals and the organic dyes was attributed to the hollow structure of the Mg/Fe-LDO nanospheres with an enhanced surface area, as well as the chemical adsorption between the anionic contaminants and the cationic Mg/Fe-LDO nanospheres through strong electrostatic interactions.

Recently, LDH-containing magnetic-hybrid nanomaterials have been explored for wastewater treatment due to their easy separation after the adsorption process [100,101,102,103]. The mechanism of the adsorption of water pollutants on magnetic LDH hybrids involves ion exchange, precipitation, surface modifications, and chelation. The incorporation of magnetic nanoparticles into the layers of LDH improves adsorption capacity, and their magnetic properties facilitate the separation of the LDH adsorbent after the adsorption process. The LDH-containing magnetic nanoparticles showed a high adsorption capacity of 262.27 mg g^−1^ for As(V) [101], 649.87 mg/g for Cr(VI) and 528 mg g^−1^ for methyl orange [102,103]. The addition of carbon-based components to the LDH layer, along with the magnetic nanoparticle Fe_3_O_4_, resulted in a novel adsorbent that can adsorb a large quantity of pollutants [104,105]. Zhang et al. prepared a Fe_3_O_4_/graphene oxide/LDH adsorbent for the removal of Pd(II) and the organic pesticide 2,4-dichlorophenoxyacetic acid from an aqueous solution [106]. The adsorption capacity of Fe_3_O_4_/LDH adsorbents for Pb(II) and the organic pollutant was substantially improved after depositing graphene oxide on the Fe_3_O_4_/LDH. In general, the carbon coating reduces the surface area, but graphene oxide contains a number of oxygen functional groups capable of binding metal ions, increasing the adsorption of ionic pollutants. Other LDH-hybrid nanomaterials including TiO_2_ and polymer-based LDHs have also exhibited enhanced adsorption behavior for water contaminants [107,108,109,110].

Despite their wide use and high adsorption ability, there are a few limitations to using nanosized inorganic nanoparticles as adsorbents. The surface energy of inorganic nanoparticles increases as their size is reduced to the nanoscale, resulting in a decrease in their stability [77,78]. As a result, they are agglomerated due to a strong van der Waals force [79,80]. After this occurs, the water treatment efficiency of the nanoparticles is dramatically decreased. To circumvent these limitations, the inorganic nanoparticles are typically incorporated into supports or other bulk adsorbents.

#### 2.2.2. Carbon Nanomaterials

Carbon-based nanomaterials, such as activated carbons, carbon fibers, carbon nanotubes (CNT), graphene, and graphene-related materials, have been considered some of the best adsorbents for removing organic and inorganic pollutants from wastewater. They exhibit high specific surface area, high porosity, excellent chemical stability in acid/alkaline conditions, and enhanced mechanical and thermal stability [111,112,113,114,115]. Despite these remarkable properties, carbon nanomaterials, particularly CNT and graphene, have limited surface functional groups and poor dispersibility in aqueous media, resulting in low adsorption [116]. Accordingly, novel carbon nanocomposite materials have been developed to improve solvent dispersibility and adsorption performance by the surface modification of CNT or graphene and the incorporation of other active materials on their surfaces [117,118,119,120].

Chemically oxidized CNTs can successfully and selectively adsorb organic dyes from wastewater through electrostatic and van der Waals interactions [121]. In addition, oxygen functional groups on the surface of CNTs can produce a partial negative charge and an electron pair to attract heavy metal ions from wastewater [122]. As a result, the oxidation of CNTs significantly enhances their adsorption capacity, allowing the selective removal of contaminants from wastewater. Yu et al. prepared oxygen-rich pentaerythritol-modified multi-walled CNTs to adsorb alizarin red S (ARS) and alizarin yellow R (AYR) from contaminated wastewater [123]. The modified CNTs showed maximum adsorption capacities of 257.73 mg g^−1^ for ARS and 45.39 mg g^−1^ for AYR. They reported that the excellent adsorption efficiency of the modified CNTs is due to the synergistic effects of the hydrogen bonding and the π–π electron stacking interactions between the adsorbents and adsorbates.

**Figure 4:**
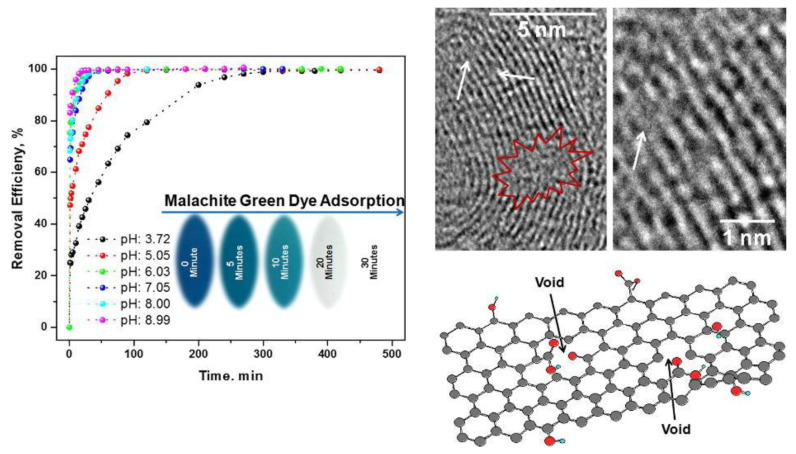
(**Left**) Effect of pH on removal of malachite green dye using rGO as an adsorbent. (**Right**) High-resolution TEM images of rGO demonstrating the holes area (encircled by red color), defect sites (indicated by white arrows), and a schematic illustration of rGO lamella consisting of holes and residual oxygen functionalities. Reproduced with permission from [131]. Copyright 2017 Elsevier.

Graphene oxide (GO) and reduced graphene oxide (rGO) have oxygen functional groups on their surface, such as hydroxyl, epoxy, and carboxyl groups, which can bond with water pollutants [124,125,126]. Major interactions between inorganic and organic adsorbates and graphene oxides involve electrostatic interactions between negatively charged graphene oxides and positively charged water contaminants. The π–π interaction between the GO ring and aromatic ring of the adsorbate was accompanied by an organic dye adsorption mechanism [127]. Many studies have reported the excellent adsorption properties of GO and rGO in regard to organic contaminants, heavy metal ions, and pharmaceutical compounds [128,129,130]. Gupta et al. studied rGO with a high surface area due to its large number of voids and pores, and demonstrated its application for the efficient removal of malachite green dye from simulated wastewater [131]. The rGO exhibited a high adsorption capacity (476.2 mg g^−1^) for malachite green dye, which was attributed to the effective π–π interaction between the aromatic moiety of the dye and the graphene skeleton, as well as the electrostatic interactions between the negatively charged oxygen functionalities of rGO and the positive center of the dye. The oxidized graphene not only increased its solubility by introducing hydrophilic oxygen species but also improved adsorption efficiency for ionic water contaminants (Figure 4). According to Zhao et al., the maximum absorption capacity of Pb (II) ions with respect to GO with few layers was determined to be 842 mg g^−1^ at 293 K, significantly greater than that of other nanomaterials [132].

Carbon nanomaterials can also be functionalized with magnetic nanoparticles to enable recovery from wastewater and their reuse after adsorption [133,134]. Magnetic metal oxide nanoparticle and carbon nanomaterial hybrids had a higher adsorption efficiency than unmodified materials, which was attributed to the active participation of the oxygen functional groups of the metal oxide in the adsorption of cationic water pollutants [135,136]. Jin et al. synthesized magnetic Ni nanoparticles encapsulated in porous carbon/CNT hybrids with a high surface area (999 m^2^ g^−1^) for the adsorption of organic dyes including malachite green, Congo red, rhodamine B, methylene blue and methyl orange, from aqueous solutions [137]. This adsorbent showed maximum adsorption capacities of 898, 818, 395, 312, and 271 mg g^−1^, respectively, resulting in significant improvements in efficiency. After the adsorption process, magnetic CNT nanohybrids can be simply separated from the solution using magnets. Kumar et al. demonstrated that the magnetic nanohybrid of GO with MnFe_2_O_4_ nanoparticles had excellent adsorption capacities of 673, 146, and 207 mg g^−1^ with respect to heavy metal ions Pb(II), As(III), and As(IV), respectively, from contaminated water [138]. They indicated that the high adsorption was attributable to a combination of the layered nature of GO, enabling maximum surface area, and the good adsorption capacities of both the GO and the magnetic nanoparticles. Jiao et al. employed nanohybrids composed of GO and magnetic nanoparticles (Fe_3_O_4_) with tunable dimensions, by an in situ deposition method for the removal of cationic dyes such as methylene blue and Rhodamine B from wastewater, and reported very high adsorption efficiencies and exceptionally high recyclability (100% removal after the 6th recycling) [139]. Koo et al. used flexible graphene-iron oxide (IO) magnetic nanosheets to purify contaminated water containing As(V) and Cr(VI) ions [140]. The IO/graphene nanosheets possess needle-like IO nanoparticles up to several hundred nanometers in length on their surfaces, which increases surface coverage. Benefiting from the high surface area, the IO/graphene nanosheets exhibited exceptional effectiveness in the removal of heavy metal ions. In addition, the superparamagnetic property of iron oxide nanoparticles facilitated recovery and recycling of the adsorbent after the adsorption process.

**Figure 5:**
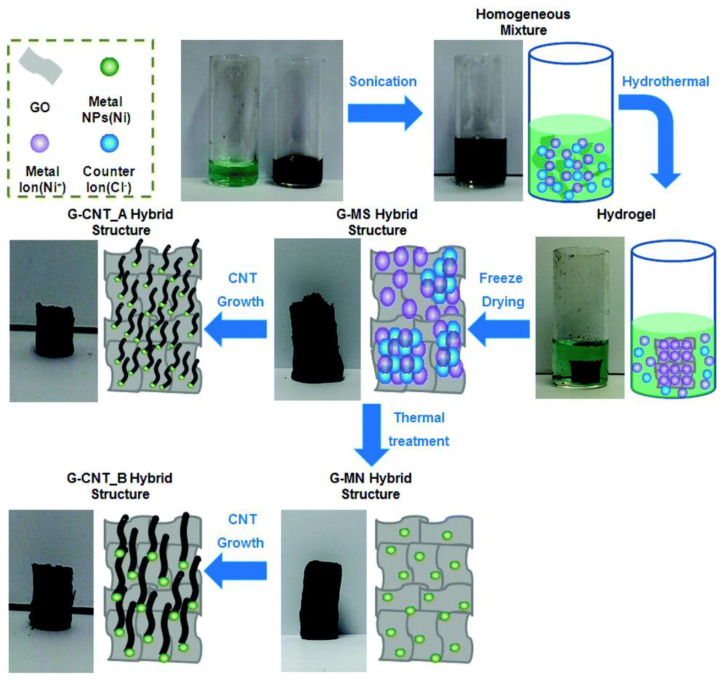
Schematic illustration of the process for fabricating the 3D graphene-CNTs hybrid structures. Reproduced with permission from [147]. Copyright 2015 Royal Society of Chemistry.

CNT and graphene aerogels have a porous structure and high specific surface area, which increases demand for their adsorption applications [141,142,143]. Ye et al. used a simple cross-linking of graphene oxide (GO) and poly(vinyl alcohol) (PVA) with glutaraldehyde to fabricate low-density (3.3 mg cm^−3^), mechanically compressible graphene aerogels for water treatment [144]. The graphene aerogels showed an ability to recover their original volume under extremely high compression stress, as well as vacuum/air-drying tolerance. By varying the mass ratio of PVA and GO, the aerogels exhibited controllable amphiphilic properties, allowing the selective absorption of hydrophilic organic dyes (methylene blue) and hydrophobic organic solvents or oils from wastewater. Yu et al. prepared hybrid aerogels composed of cellulose nanofibrile and carbon nanomaterials, CNTs, and graphene, which demonstrated high adsorption of cationic (methylene blue) and anionic (Congo red) organic dyes [145]. According to their results, the maximum adsorption capacities were 1178.5 mg g^−1^ and 585.3 mg g^−1^ for methylene blue and Congo red, respectively. Generally, CNTs and graphene together have high mechanical strength and elasticity, whereas aerogels comprised either of neat CNT or graphene exhibit relatively low elasticity [146]. To address this drawback, research has been carried out in on combining graphene and CNTs to prepare three-dimensional aerogels as adsorbents. Incorporating CNTs into graphene aerogels improved more than 100-fold the adsorption efficiency of the nanohybrid compared with the graphene aerogel [146]. Lee et al. employed CNT–graphene nanohybrid aerogels to remove organic contaminants such as methyl orange, methylene blue, Congo red, and crystal violet from wastewater [147]. In the nanohybrid, CNTs bound to the graphene aerogel, resulting in considerable improvement in mechanical and electrical properties, as well as a 57% increase in the surface area of graphene aerogel. The CNT–graphene nanohybrid aerogel successfully adsorbed both the anionic and cationic organic dyes with significant efficiency, through π–π and van der Waals interactions (Figure 5). Ai et al. synthesized a self-assembled cylindrical CNT–graphene hybrid by a one-step hydrothermal process for the removal of methylene blue, and observed a maximum adsorption capacity of 81.97 mg g^−1^ [148]. In another study, Wan et al. prepared a CNT–graphene aerogel by a hydrothermal redox reaction [149]. According to morphology studies using FE-SEM and TEM, large numbers of entangled CNTs tightly cover the graphene sheets and the CNTs are networking graphenes. The aerogel had ultra-light densities in the range of 6.2 to 12.8 mg cm^−3^, with an improved specific surface area and improved mechanical properties. It exhibited excellent adsorption capacity with respect to methyl blue, methyl orange, and oil species.

**Figure 6:**
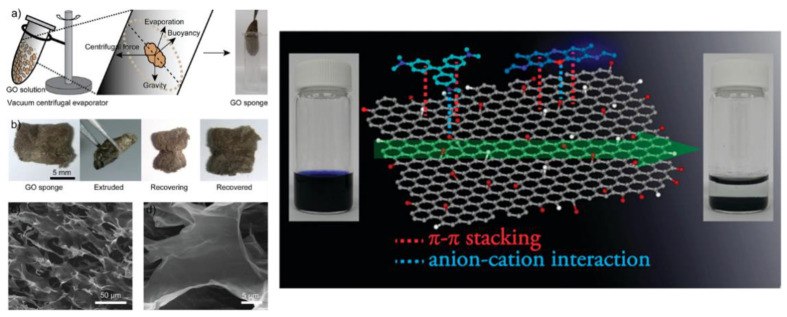
(**Left**) (**a**) Synthetic scheme of a 3D GO sponge. (**b**) Flexibility test of a GO sponge. (**c**) Low-magnification of SEM image for the GO sponge surface and (**d**) high-magnification of SEM image for the inner part of the 3D GO sponge. (**Right**) Chemical structures of methylene blue, methyl violet, and GO. Digital images of the original MB dye solution, the pale color solution with precipitated MB adsorbed GO sponges. Reproduced with permission from [159]. Copyright 2012 American Chemical Society.

Despite having various advantages as an adsorbent, carbon nanomaterial has several disadvantages including difficulties in segregating nanoadsorbents from water, limited adsorption capacities, and fouling effects. These problems can be solved by the development of multifunctional macroscopic three-dimensional (3D) architectures, using carbon nanomaterials as building blocks. The construction of carbon nanomaterials into three-dimensional 3D structures has attracted attention for various applications, including adsorption, because the carbon nanomaterial maintains the intrinsic properties of its 1D or 2D precursor while overcoming its drawbacks [150,151,152]. The existing research on 3D carbon-based materials for adsorption relates mostly to graphene-based nanomaterials. 2D graphene-based materials can be fabricated into 3D graphene-based materials by using template-oriented building methods, self-assembly methods, and 3D printing methods [153]. Graphene aerogels, hydrogels, sponges, and foams are examples of 3D graphene-based nanoadsorbents developed and applied to adsorption, exhibiting outstanding adsorption capabilities [154,155,156]. Their larger pore volumes, high surface areas, controlled geometries, extra-light densities, and strong mechanical stabilities have made 3D graphene-based materials promising candidates for environmental remediation adsorbents. In 3D graphene-based nanoadsorbents, major driving forces for the adsorption of pollutants involve π–π interactions, hydrogen bonding, electrostatic interactions, ion-exchange, and surface complexation [157,158]. Liu et al. prepared 3D GO sponges from a GO suspension by a simple centrifugal vacuum evaporation method. These 3D GO sponges exhibited adsorption capacities of 397 and 467 mg g^−1^ for methylene blue and methyl violet dyes, respectively (Figure 6) [159]. Sui et al. reported the fabrication of 3D GO-PEI (polyethyleneimine) porous foams with bulk densities in the range of 0.02–0.03 g cm^−3^, with adsorption capacities of 800 mg g^−1^ with respect to acidic dyes such as amaranth [160]. Jayanthi et al. prepared ultra-light and highly porous 3D GO foams by a lyophilization method. These were used for the removal of three carcinogenic dyes, ie., rhodamine B, malachite green, and acriflavine, and for the degradation of *E. coli* bacteria [161]. For the three dyes, the 3D GO foams exhibited adsorption abilities of 446, 321 and 228 mg g^−1^, respectively. In another study, CNTs were incorporated into a polyacrylamide-sodium alginate-based polymer hydrogel, which exhibited a macroporous structure with a very low density. The adsorption capacity of the CNT–polymer hydrogel was 1.28 times higher than that of a polymer hydrogel without CNT, showing 38.9 mg g^−1^ with respect to Cu^2+^ ions [162].

The 3D networks integrate the macroscale and nanoscale architectures of the carbon nanomaterial building blocks, preserving their intrinsic properties while introducing to the carbon nanomaterials unique features such as easy recycling, additional binding sites, and simple operation. Meanwhile, problems associated with these structures are instability and fragility [117]. The mechanical properties of the structures could be enhanced by surface modification with nanofillers or nanofibers, improving cross-linking conditions, and by incorporation of the polymer matrix. Moreover, further effort is needed to improve our understanding of growth mechanisms to produce carbon nanostructured materials that are stable, shape-controllable, and inexpensive.

#### 2.2.3. Biopolymer Adsorbents

**Figure 7:**
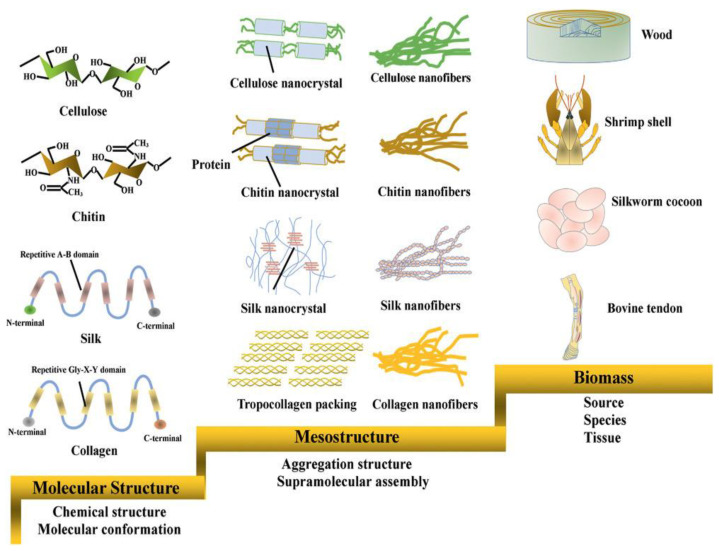
Hierarchical structures of cellulose, chitin, silk fibroin, and collagen in wood, shrimp shell, silkworm cocoon, and bovine tendon. Reproduced with permission from [163]. Copyright 2021 John Wiley and Sons.

Biopolymers (or natural polymers) have been an area of focus in the treatment of wastewater pollutants because of their sustainability, biodegradability, availability, and biocompatibility [117,163,164]. Biopolymers include chemically varied macromolecules such as polysaccharides, polypeptides, polynucleotides, polyesters, and polyaromatics, found in plant- and microbe-derived cellulose, fungus-derived chitin, animal-produced silk, and mammal-derived collagen (Figure 7). The biopolymers most commonly used in the adsorption of water contaminants are cellulose, chitosan, chitin, and lignin [117].

Nanocellulose-based materials have been widely studied for environmental remediation due to their low cost, ecological sourcing and non-toxicity [163]. Generally, the primary types of nanocellulose include cellulose nanocrystals, cellulose nanofibrils, and bacterial cellulose (BC), each with their own production process and morphology [165]. Nanocellulose-based materials can be used as absorbents, membrane filters, and composites for water treatment as well as air purification. In order to further improve adsorption capability for organics and heavy metal contaminants, the surface of nanocellulose was modified with various functional groups [163,165]. Hokkanen et al. prepared succinic anhydride-modified nanocellulose to adsorb Zn(II), Ni(II), Cu(II), Co(II), and Cd(II) ions, demonstrating significant efficiency and recycling properties [166]. Maatar et al. used highly porous cellulose organogels from nanofibrillated cellulose hydrogels as adsorbents of organic pollutants such as aromatic compounds and herbicides [167]. Chan et al. synthesized cellulose nanofibrils via an acidified-chlorite bleaching method and used them for methylene blue adsorption [168]. The maximum adsorption capacity was 123 mg g^−1^ at 20 °C and pH=9. Cellulose nanocrystals oxidized with TEMPO(2,2,6,6-tetramethylpiperidine 1-oxyl) had increased adsorption capacity (769 mg g^−1^) with respect to methylene blue [169]. In another study, amino-functionalized nanocrystalline cellulose exhibited excellent absorption capacity with respect to Congo red 4BS, acid red GR, and reactive light-yellow K-4G, at up to 200, 138.9, and 188.7 mg g^−1^, respectively [170].

Chitosan, a main derivative of chitin that can be achieved by the bioconversion of chitin using deacetylation, is an important natural polymer recognized for its biodegradability and biocompatibility, non-toxicity, and outstanding adsorption capabilities [171]. Many chemical modifications have been performed on chitosan and chitin for specific environmental application [172,173]. Chitosan, chitin, and their derivative molecules have been used as natural and environmentally friendly coagulants/flocculants to remove various charged particles from wastewater, including heavy metal ions and organic dyes. Polyethylene glycol/chitosan and polyvinyl alcohol/chitosan composites were shown by Rajeswari et al. to remove aqueous nitrate ions with adsorption capacities of 50.68 and 35.03 mg g^−1^, respectively [174]. Borsagli et al. used carboxymethyl-chitosan for the adsorption of Cd(II) and Cr(VI) ions [175], while Rahimi et al. used goethite/chitosan nanocomposite for the removal of Pb(II) ions [176]. Gibbs et al. studied the sorption of acid green 25 by chitosan, varying experimental parameters such as pH and the chitosan adsorbent particle size [177]. They reported a maximum adsorption capacity of 700 mg g^−1^ in the pH range of 2–4. Marrakchi et al. synthesized a cross-linked chitosan/sepiolite composite from chitosan and sepiolite clay, a porous zeolite-like mineral [178]. The composite was used as an adsorbent and showed a maximum absorption capacity of 40.99 mg g^−1^ and 190.97 mg g^−1^ for methylene blue and orange 16, respectively.

Chitosan-based nanomaterials have been investigated for their ability to remove dye molecules, with hydroxyl groups efficiently utilized in dye adsorption while the amine groups firmly remain the most active group and affect other biopolymer activities [179,180]. Gopi et al. fabricated multi-functional hybrid bio-aerogels for wastewater treatment, composed of chitin nanocrystals and cellulose nanofibers [181]. The hybrid bio-aerogels showed a considerable adsorption capability for the extraction of dyes (methylene blue and rhodamine 6 G) from aqueous solutions. They reported that dye adsorption was improved by the electrostatic interactions between positively charged dyes and negatively charged chitin nanocrystals containing acetamide-enriched groups. Furthermore, the aerogels showed antibacterial and antioxidant activity against four bacterial species, including *E. coli*, *S. typhimurium*, *S. aureus*, and *B. cereus*. Zhang et al. prepared a dialdehyde microfibrillated cellulose/chitosan composite film with a 3D network of microcrystalline cellulose for the uptake of Congo red. The adsorption capacity for Congo red was 152.5 mg g^−1^ with 99.95% removal efficiency within 10 min of contact time [182].

**Figure 8:**
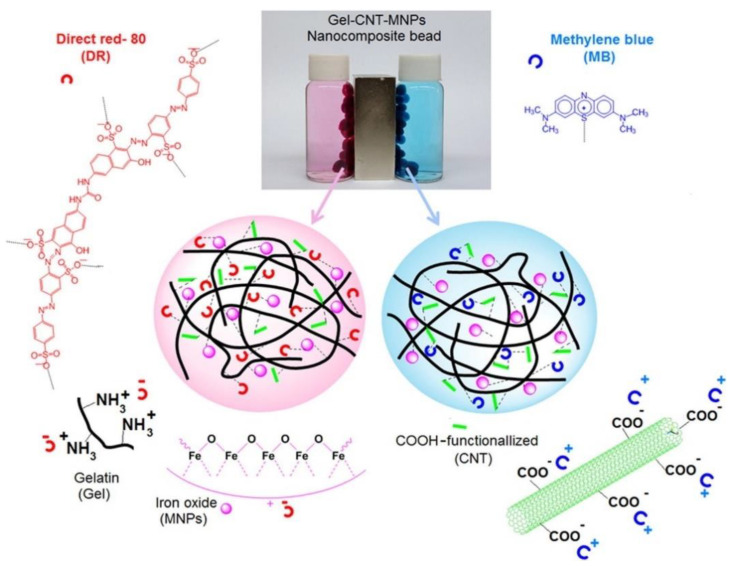
Schematic illustration for the adsorption of direct red-80 and methylene blue by the prepared nanocomposite bead, the digital image of the nanocomposite bead (Gel-CNT-MNPs) immersed in the dyes solutions in the presence of magnet. Reproduced from [193] with permission. Copyright 2017 Elsevier.

Although biopolymers have the potential to be used as pollutant adsorbents due to their environmental and economic advantages, the contaminant adsorption rate of biopolymers is not commercially practical. This is because water molecules reduce the affinity between the dye and the biopolymer adsorbent in aqueous environments, interfering with the adsorption process [183,184]. Furthermore, limitations for practical application also include the difficulty in recovering biopolymers after contaminant adsorption and their poor mechanical strength [185]. Therefore, biopolymers are often integrated with carbon-based nanomaterials to improve their mechanical properties and chemical stabilities; at the same time, they stabilize the carbon-based nanomaterial to prevent agglomeration [186]. The incorporation of chitosan into graphene is the most popular method of achieving mutual stabilization and improvement during the adsorption process [187,188,189]. Dai et al. prepared polyvinyl alcohol (PVA)/carboxymethyl cellulose hydrogels reinforced with GO and bentonite [190]. The carboxymethyl cellulose was isolated from pineapple peel, and the hydrogel was prepared using freeze–thaw cycles. The introduction of GO and bentonite to the hydrogels improved their thermal stability, swelling ability, and adsorption capacity for methylene blue dye. By introducing these two fillers, the calculated maximum adsorption capacity of the hydrogels increased to 172.14 mg g^−1^ from 83.33 mg g^−1^. Li et al. successfully synthesized a lignin-grafted CNT nanocomposite to remove Pb(II) to obtain significant adsorption capacity (235.0 mg L^−1^) [191]. They reported that the high adsorption efficiency was due to the nanocomposite’s 3D structure, high surface area, large number of oxygen functional groups, large pore size, and mechanical stability. Gao et al. reported a one-step synthesis of a polydopamine-functionalized graphene hydrogel for the adsorption of heavy metal ions (Pd(II) and Cd(II)), an organic dye (rhodamine B), and an aromatic contaminant (p-nitrophenol) [192]. Saber-Samandari et al. recently described gelatin–CNT–iron oxide magnetic nanocomposite beads for the adsorption of anionic (direct red 80) and cationic (methylene blue) dye from aqueous solutions (Figure 8) [193]. The magnetic biosorbent removed both dyes with high adsorption efficiency (96.1% for direct red 80 and 76.3% of methylene blue) and was easily isolated with the help of an external magnet.

#### 2.2.4. Others

Nanoporous materials, particularly well-ordered mesoporous ones, are recognized as excellent adsorbents due to their high surface areas and their large and regularly structured mesoscale channels, allowing fast mass transfer kinetics [194,195,196,197]. However, adsorption efficiency depends not only on surface area to supply active adsorption sites and pathways for the quick approach of adsorbates, but also on the interaction between the active adsorption sites and the targeted adsorbates, which controls the strength and the selectivity of the adsorption [198,199]. Therefore, in addition to pore structure engineering, chemical modification of the surface is required in the design of nanoadsorbents for wastewater treatment. Examples include mesoporous silica materials functionalized with thiol-based functional groups (thiol, thiourea, and thioether) on their pore surfaces for the removal of mercury ions [199,200,201]. Li et al. synthesized a thiol-functionalized, porous organic polymer-based nano-trap for the removal of Hg(II) ions [202]. The nano-trap exhibited a record-high saturation Hg(II) adsorption capacity of over 1000 mg g^−1^, able to reduce Hg(II) concentration from 10 ppm to a level appropriate for potable water (0.4 ppb, WHO standards) within a few minutes. More importantly, the nano-trap showed high stability and adsorption efficiency over a broad pH range owing to its stable C–C bond, and it remained stable at temperatures up to 270 °C.

An aptamer is a newly developed type of ligand and short single-stranded oligonucleotide with a highly specific and strong affinity for a target molecules [203,204]. A special aptamer can be selected via the systematic evolution of ligands by exponential enrichment (SELEX) for specific target molecules, from a comprehensive library of DNA molecules containing randomly created sequences [205,206]. This strategy has been used in a variety of research areas, including nanomaterial synthesis and sensor development, and has recently been used in water purification [207,208]. Kim et al. selected single-stranded DNA aptamers from a random library for arsenate [As(V)] and arsenate [As(III)] binding via SELEX. The selected aptamers had an extraordinarily high affinity for both As(V) and As(III), with dissociation constants (*K*_d_) of 4.95 and 7.05 nM, respectively [207].

Similar to the aptamer-based strategy, molecular imprinting techniques have shown a high affinity and selectivity towards the template molecule even in the presence of interfering substances [209,210]. One notable study on molecular imprinting for water treatment involved the fabrication of core–shell structured nanocomposites, consisting of a magnetic nanoparticle core and a molecular-imprinted polymer shell, for the selective adsorption of water contaminants. Li et al. recently reported the synthesis of core–shell molecularly imprinted magnetic polymer beads by reversible addition–fragmentation chain transfer polymerization [211]. The core–shell magnetic beads were utilized for the recognition and separation of endocrine disrupting chemicals, such as Bisphenol A, from aqueous solutions.

**Figure 9:**
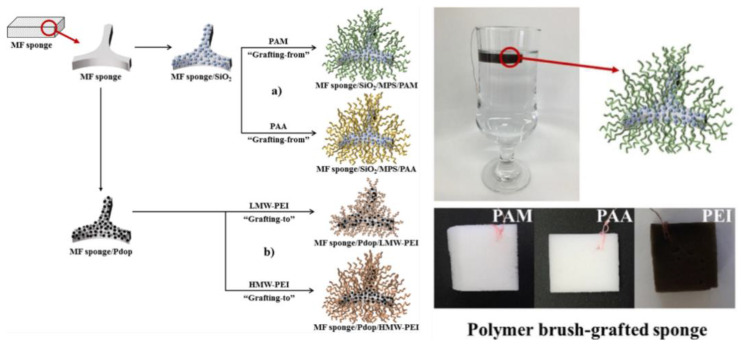
(**Left**) Schematic illustration for the fabrication of melamine formaldehyde (MF) sponge composites. The PAM and PAA brushes were grafted by the “grafting-from” method, and PEI was grafted by the “grafting-to” method. (**Right**) Image of a glass of water containing a low concentration of each heavy metal ion (Cu^2+^: 0.368 mg/L or Pb^2+^: 0.250 mg/L) after submerging a PEI-coated sponge (upper image). Images of various polymer brush-grafted sponge. Reproduced from [222] with permission. Copyright 2016 Elsevier.

A polymer brush is a layer of polymer chains attached to a surface by the end of the polymer chain, a technique that facilitates an increase in the density of surface functional groups [212,213,214,215,216]. On the surface of porous adsorbents, functional groups are typically limited to a single layer incorporated onto the adsorbent surfaces with only a few functionalities per molecule. Polymer brushes possess multiple repeating functional groups and can be firmly bonded to the surface of a wide range of materials using covalent bonds. Several research groups have designed cationic or anionic polymer brush-grafted magnetic nanoparticles for highly efficient water remediation [216,217,218,219,220,221]. Bae et al. developed polymer-grafted sponges composited with various polyelectrolyte brushes, such as polyacrylamide (PAM), polyacrylic acid (PAA), and poly polyethyleneimine (PEI), and used them as portable heavy metal ion (Cu(II) and Pb(II)) adsorbents [222]. To control the length of the polymer brushes related to the concentration of active functional groups, the authors employed polyelectrolytes with varying molecular weights and evaluated the influence of the length of the polymer brush on adsorption performance (Figure 9). According to their results, the low-molecular-weight PEI-grafted sponges showed fast removal ability with respect to heavy metal ions, and the high-molecular-weight PEI-grafted sponges showed the highest adsorption capacities (3.922 mmol g^−1^) due to their abundant adsorption sites. These PEI-grafted sponges demonstrated no significant cytotoxic activity in relation to cells. By simple immersion, they completely purified a glass of water contaminated with a low concentration of heavy metal ions, proving easy application by individual users.

Nanofibers are fibrous materials with nano-scaled diameters that have received tremendous attention due to their promising properties and characteristics for a variety of applications. Polymer nanofiber membranes have emerged as attractive adsorbents for wastewater treatment applications because of their easy surface modification, high flexibility, high porosity, and easy separation [223,224,225,226]. Polymer nanofiber membranes produced by electrospinning have been the object of increasing attention over the past decade, and the technique has been extensively studied in terms of its design, mechanism, applications, and technical issues [227,228,229]. The electrospinnun polymer nanofiber membranes have a high aspect ratio and surface adsorption energy, providing an effective adsorbent material that can be regenerated without secondary contamination [230,231,232,233]. Recently, Zhao et al. reported a simple strategy to fabricate amine-functionalized ultrathin polyacrylonitrile nanofiber of 100 nm diameter using a mixed solution electrospinning procedure, to efficiently remove organic dyes from aqueous media [234]. The ultrathin polyacrylonitrile nanofiber membrane possesses abundant amino groups on the surface, contributing to its ultrahigh adsorption capacity of more than 1800 mg g^−1^ and rapid adsorption rate with concentrations ranging from 20 to 100 mg L^−1^ for organic dye, Direct Red 23. Park et al. fabricated nanofibrous adsorbents by electrospinning with a blend solution of poly(acrylic acid) and poly(vinyl alcohol) polymers and used these for Cu(II) removal from industrial plating wastewater. The nanofibrous adsorbents had a maximum removal capacity of 49.3 mg g^−1^ for Cu(II) ions with higher selectivity for Cu(II) over Ni(II) in a binary system [235].

Metal–organic frameworks (MOFs) are highly ordered crystalline coordination polymers with the advantages of large specific surface area, tunable pore size, high porosity, outstanding absorbability, and controlled modification synthesis [236,237,238]. Those advantages are highly favourable to adsorption and separation [239,240,241] and have been widely utilized to remove toxic contaminants, such as organic dyes or heavy metal ions in aqueous media [242,243,244].

Dendritic polymers are novel nanostructured synthetic polymers exhibiting a highly branched structure with unique three-dimensional architecture and numerous active functional groups [245,246,247,248]. Since dendritic polymers are relatively cheap, less toxic, easy to functionalize over other substrates, and highly efficient, they also have been widely employed as adsorbents for removal of inorganic and organic pollutants from water [249,250].

## 3. Summary and Perspective

Hazardous pollutants in wastewater, such as heavy metal ions and organic dyes, pose a serious environmental threat. Wastewater treatment before disposal and recovery is a high priority, and it is a challenge to develop a general approach for removing all types of contaminants from wastewater. In this regard, adsorption technology is well established in the industry and has been shown to remove many kinds of contaminants from wastewater. In recent decades, there have been significant efforts to design and produce highly efficient and environmentally friendly adsorbents for wastewater treatment. Due to their unique physicochemical properties, nanostructured adsorption materials have received intensive attention for their application in the adsorptive removal of various contaminants in wastewater. This review summarizes recent advances in the preparation and adsorption performance of nanostructured materials used for the removal of contaminants in wastewater.

Inorganic-based metal oxides and carbon nanomaterials have been widely studied and frequently used as adsorbents for the removal of toxic pollutants in aqueous solutions. Hierarchical structures, such as core–shell, layered, hollow, and flower-shaped nanoparticles, have been designed to enhance the removal efficiency of adsorbents. The ability of an adsorbent to regenerate and to be reused is important in terms of practicality and economic cost. The incorporation of magnetic nanoparticles into an adsorbent has demonstrated effective regeneration. Despite their wide use and high adsorption ability, metal oxide nanoparticles and carbon nanomaterials each have technical drawbacks. For instance, reducing the size of particles into the nanoscale can increase their surface area, but it can cause instability in an aqueous solution and aggregation into large-size particles. As a result, their adsorption efficiency is inevitably reduced. In addition, the isolation of used nanomaterials from water in an efficient and cost-effective manner remains a challenge. Inability to isolate can affect the mobility and bioavailability of toxic pollutants in the environment, and this may increase the toxicity of the nanomaterials and cause secondary pollution in any surroundings.

Biopolymer- and polymer-based nanomaterials have been considered candidates for the ecological treatment of wastewater pollutants. Because of their unique structural and physical properties, their availability, safety, and cost, (bio)polymer-based nanomaterials offer a variety of uses for the generation of sustainable materials; the biodegradability and biocompatibility of natural resources can improve their utilization as nanosorbents. However, though (bio)polymers have the potential to be used as pollutant adsorbents due to their environmental and economic advantages, contaminant adsorption using biopolymer is not commercially practical. This is due to the low affinity between pollutants and polymer adsorbents in aqueous solutions, resulting in low adsorption ability. Other limitations to practical applications include the difficulty of recovering the biopolymer after contaminant adsorption, and its poor mechanical strength. Therefore, (bio)polymer is often integrated with metal oxide and carbon-based nanomaterial to improve its mechanical properties and chemical stability.

Although adsorption using nanostructured materials is a promising approach for wastewater purification, it generates secondary environmental toxic waste. It is essential to mitigate this problem sustainably. It may be solved by reusing spent materials in various applications, including catalysis, sensors, and energy applications [251,252]. Future study will be required to add value to secondary toxic waste through recyclability and circular strategy. Despite several limitations, nanostructured adsorbents have revealed their effectiveness in removing toxic pollutants from water and wastewater. The use of nanoadsorbents to remove pollutants, addressing the limitations of recycling and separation, has become a practical and easy process. However, it is still in its early stages, and various challenges need to be addressed, including the development of easier processes to obtain efficient nanoadsorbents as well as their field application in water and wastewater treatment. Moreover, strong collaboration between industry and academia is required to develop and employ novel wastewater treatment technologies as soon as they become available.
